# External validation of a 3-step falls prediction model in mild Parkinson’s disease

**DOI:** 10.1007/s00415-016-8287-9

**Published:** 2016-09-19

**Authors:** Beata Lindholm, Maria H. Nilsson, Oskar Hansson, Peter Hagell

**Affiliations:** 1 Department of Neurology and Rehabilitation Medicine, Skåne University Hospital, Malmö, Sweden; 2 Clinical Memory Research Unit, Department of Clinical Sciences, Lund University, Malmö, Sweden; 3 Department of Health Sciences, Lund University, Lund, Sweden; 4 Memory Clinic, Skåne University Hospital, Malmö, Sweden; 5 The PRO-CARE Group, School of Health and Society, Kristianstad University, Kristianstad, Sweden

**Keywords:** Parkinon disease, Falls, Near falls, Gait, Balance, Prediction

## Abstract

The 3-step falls prediction model (3-step model) that include history of falls, history of freezing of gait and comfortable gait speed <1.1 m/s was suggested as a clinical fall prediction tool in Parkinson’s disease (PD). We aimed to externally validate this model as well as to explore the value of additional predictors in 138 individuals with relatively mild PD. We found the discriminative ability of the 3-step model in identifying fallers to be comparable to previously studies [area under curve (AUC), 0.74; 95 % CI 0.65–0.84] and to be better than that of single predictors (AUC, 0.61–0.69). Extended analyses generated a new model for prediction of falls and near falls (AUC, 0.82; 95 % CI 0.75–0.89) including history of near falls, retropulsion according to the Nutt Retropulsion test (NRT) and tandem gait (TG). This study confirms the value of the 3-step model as a clinical falls prediction tool in relatively mild PD and illustrates that it outperforms the use of single predictors. However, to improve future outcomes, further studies are needed to firmly establish a scoring system and risk categories based on this model. The influence of methodological aspects of data collection also needs to be scrutinized. A new model for prediction of falls and near falls, including history of near falls, TG and retropulsion (NRT) may be considered as an alternative to the 3-step model, but needs to be tested in additional samples before being recommended. Taken together, our observations provide important additions to the evidence base for clinical fall prediction in PD.

## Introduction

Falls and balance problems are common already early in Parkinson’s disease (PD) [1–4] and progress over time [5–7]. Avoiding falls and its consequences is a major goal and challenge in the management of PD [8]. Several predictive factors for future falls and near falls have been identified, e.g. history of falls and near falls, impaired balance, retropulsion, reduced comfortable gait speed, freezing of gait (FOG), cognitive impairments, pain, and fear of falling (FOF) (e.g. [9–12]). However, prediction of falls is still a clinical challenge. For example, most available studies have identified predictive factors based on logistic regression models and associated odds ratios (ORs) (e.g. [10–12]). However, ORs do not inform about the ability of predictors to discriminate between future fallers and non-fallers [13, 14] and are therefore not easily implemented in clinical practice [15]. It is therefore unclear exactly what components to consider. For example, history of falls was proposed as the strongest predictor in several prospective studies [16], whereas other observations suggest that history of near falls is a stronger predictor and may be seen as a precursor of falls [12].

Successful implementation of prediction models in clinical practice generally requires three main phases: model development, external validation, and investigations of their clinical impact; it is also recommended to consider whether existing models can be improved by, e.g. additional predictors [17]. Furthermore, in order to be applicable, useful and practical for routine clinical use, prediction models need not only to have sufficient ability to discriminate between future fallers and non-fallers, but also be easy and quick to implement. To this end, Paul et al. [18] proposed a 3-step falls prediction model (3-step model) consisting of three variables: history of falls during the past 12 months, history of FOG, and comfortable gait speed <1.1 m/s based on the mean of two trials of walking a standardized distance. Receiver operating characteristics (ROC) curve analyses found the 3-step model to discriminate between fallers and non-fallers over a prospective 6-month period with an area-under-the-curve (AUC) of 0.80 (95 % CI 0.73–0.86) [18]. These results were later replicated in a different PD sample (AUC, 0.83; 95 % CI 0.76–0.89) [19].

In contrast to other suggested fall prediction models, the 3-step model avoids reliance on relatively lengthy and time consuming clinical tests and assessments. For example, the model proposed by Kerr et al. [9] involves total scores of the Unified PD Rating Scale (UPDRS), the Tinetti scale and the FOG Questionnaire (FOGQ). The 3-step model therefore appears clinically promising and has been recommended in the European Physiotherapy Guidelines for PD [20]. However, it may be argued that measurement of gait speed according to the 3-step model may be considered cumbersome or difficult to achieve in clinical practice since it requires a standardized distance free of narrow passages, timing of two walking trials, and the calculation of the corresponding mean velocity as m/s. Meanwhile, common and easily conducted clinical PD tests such as pull-tests [21–24] and Tandem Gait (TG) [23, 25] were not considered in the development of the 3-step model [18], although previously recommended for the prediction of falls [23, 24].

This study aimed to externally validate the 3-step model in an independent sample of people with relatively mild PD. In addition, we explored the ability of additional historical information and clinical tests to predict falls as well as near falls, and compared those with the proposed 3-step model.

## Method

### Participants

The Regional Ethical Review Board approved the study (Dnr 2011/768). All participants gave written informed consent.

Participants were enrolled in a cohort study designed for evaluation of a broad spectrum of factors associated with falls and near falls in PD. All people diagnosed with PD receiving care at a south Swedish university hospital during 2007–2013 were considered eligible for inclusion (*n* = 359). Exclusion criteria were: age above 80 years old (*n* = 121), inability to understand instructions (*n* = 14), significant cognitive impairment (Mini-Mental State Examination (MMSE) score <24; *n* = 8), inability to stand without support (*n* = 22) and severe comorbidity (*n* = 11). Of the remaining 183 potential participants, 40 (16 women) declined participation and 5 did not complete the follow-up period, leaving 138 participants in the final study sample.

### Assessments and procedure

Detailed description of the procedures are available elsewhere [12]. All participants were assessed during an outpatient visit, scheduled at a time of day when the participant usually reported to feel at best.

Data for the proposed 3-step model [18] were taken from the following sources: (1) history of falls was determined by yes/no responses to the question: *In the last 12* *months, have you fallen in such a way that your body hit the ground?* In addition, history of near falls was considered (but not as part of the proposed 3-step model; see below) by responses to a similar yes/no question: *Are you ever close to falling, but you manage to grab on to something/someone at the last minute so that your body does not hit the ground?*; (2) responses to item 3 [*Do you feel that your feet get glued to the floor while walking, making a turn or when trying to initiate walking (freezing)?*] of the FOGQ (self-administered version) [26], which is scored 0–4 (higher = worse); those scoring ≥1 were categorized as having history of FOG [27]; (3) gait speed measurement according to the 10-Meter Walk test (10MWT), conducted in comfortable gait speed without acceleration, using a digital timer (Origo, model 365510). To ensure the relevance of the suggested 1.1 m/s cut-off [18] we tested the optimal cut-point in the current sample [28], which was found to be 1.06. We therefore calculated each person’s mean m/s from two trials and dichotomized the resulting mean m/s according to the proposed 1.1 m/s cut-off.

Retropulsion was assessed using an unexpected shoulder pull according to the Nutt Retropulsion test (NRT) [22] as well as an expected shoulder pull according to item 30 of the UPDRS [21]. The participant was standing with feet slightly apart and eyes open, with the examiner giving a sudden, firm backward pull to the shoulders from behind. The NRT was executed first and scored 0-3: 0 (normal, ≤2 steps to recover), 1 (≥3 or more steps; recovers unaided), 2 (would fall if not caught), 3 (spontaneous tendency to fall or unable to stand unaided) [22, 24]. Those scoring ≥1 were categorized as having retropulsion [24]. During assessments according to UPDRS item 30, the participant was first told that s/he was to be pulled and instructed to prevent falling [21, 29]. Performance was scored 0–4: 0 (normal), 1 (retropulsion, but recovers unaided), 2 (absence of postural response, would fall if not caught by examiner) and 3 (very unstable, tends to lose balance spontaneously), 4 (unable to stand without assistance). Those scoring ≥1 were categorized as having retropulsion [24].

To assess the ability to walk in tandem (TG) participants were instructed to take ten consecutive heal-to-toe steps along a straight line without walking aids or support, with eyes open. Performance was scored 0–3; 0 (no side steps), 1 (a single side step), 2 (multiple side steps), 3 (unable to take 4 consecutive steps) [30]. Those scoring ≥1 were categorized as having an abnormal TG performance [23, 25].

Additionally, demographic data (age, gender, PD duration and severity according to Hoehn and Yahr [HY]) [31] were recorded and parkinsonian motor symptoms were assessed using the UPDRS part III (motor examination), which yields a total score ranging from 0 to 108 (108 = worse) [21]. Antiparkinsonian medications were recorded from medical records and expressed as daily levodopa equivalent (LDE) doses (mg/day) [32].

Finally, participants were provided with a diary for recording prospective falls and near falls during a six-month follow-up, where falls were defined as “*an unexpected event in which the participants come to rest on the ground, floor, or lower level*” [33], and near falls were defined as “*a fall initiated but arrested by support from the wall, railing, other person* etc.” [34]. In the diary, two yes/no questions were used to define whether an incidence was a fall (*Did you fall in such a way that your body hit the ground?*) or a near fall (*Were you close to falling, but managed to brace yourself at the last moment (e.g. grabbed on to someone, to an object or the wall?*) [12]. Those reporting at least one fall or near fall were considered fallers and near-fallers, respectively. To facilitate correct registration during the 6-month follow-up the definitions of a fall and a near fall were thoroughly described to all participants at the outpatient visit. All participants were also telephoned monthly to ensure that registrations were completed according to instructions. During the last telephone call, they were requested to return the diary in a pre-stamped envelope.

### Statistical analyses

Data were checked regarding underlying assumptions and analysed accordingly using IBM SPSS version 22 (IBM Corp., Armonk, NY). The alpha level of significance was set at 0.05 (two-tailed).

To externally validate the 3-step model [18, 19] multiple logistic regression analysis (enter method) was used with the three suggested predictors (history of falls in 12 months, history of FOG, and comfortable gait speed <1.1 m/s) as independent variables and occurrence of falls during the 6-month follow-up as the dependent variable. In developing the 3-step model, Paul et al. [18] suggested weights for each predictor variable based on the ORs from their logistic regression model (history of falls, weight 6; history of FOG, weight 3; gait speed <1.1 m/s, weight 2), yielding a summed total score between 0 and 11. Based on these, three risk categories and associated 6-month prospective fall probabilities were suggested: low risk (score 0; 17 % fall probability), moderate risk (score 2–6; 51 % fall probability), and high risk (score 8–11; 85 % fall probability) [18]. In order to facilitate comparisons with prior studies [18, 19], risk categories for our sample were derived by the sum of the suggested predictor weights.

Secondly, simple logistic regression analyses were used to evaluate how well each single predictor (history of falls in 12 months, history of near falls, history of FOG, gait speed, NRT, UPDRS item 30, and TG) predicted falls during the 6-month follow-up. Thirdly, all potential predictors were entered into a multiple logistic regression analysis (backward method) to explore if this could improve prediction of future falls as compared to the 3-step model. We also calculated the sensitivity and specificity of relevant prediction models. In order to account for near falls, we also explored factors associated with the combination of prospective falls and/or near falls according to the same procedures as described above.

ROC curve analyses were used to assess overall accuracy of each model by estimating the AUC [35, 36]. AUCs can range between 0 and 1; an AUC <0.5 indicates that the model performs worse than chance, whereas an AUC of 1 indicates perfect discrimination. AUCs ≥0.7 and >0.9 are considered acceptable and high, respectively [37].

## Results

The final sample (*n* = 138) is summarized in Table 1. During the 6 months of follow-up, 33 % (45/138) reported ≥1 fall and 14 % (19/138) reported only near falls (Table 2).

**Table 1:** Sample characteristics, *n* = 138

Age (years), mean (SD; min–max)	67 (9.8; 35–80)
Female gender, *n* (%)	64 (46)
History of falls, *n* (%)^a^	38 (28)
History of near falls, *n* (%)^b^	48 (35)
History of FOG, *n* (%)^c,d^	57 (41)
Severity of disease (H&Y), median (q1–q3; min–max)	2 (2–3; 1–4)
PD-duration (years), mean (SD; min–max)	4 (4.0; 0.1–17)
Motor symptoms (UPDRS part III), median (q1–q3; min–max)	12 (8–18; 1–34)
Comfortable gait speed <1.1 m/s (10MWT), *n* (%)^e^	54 (39)
Retropulsion (NRT), *n* (%)^f^	36 (26)
Retropulsion (UPDRS, item 30), *n* (%)^g^	53 (38)
Abnormal tandem gait (TG), *n* (%)^h^	78 (57)
Daily total levodopa equivalent (LDE) dose (mg), median (q1–q3; min–max)^i^	400 (300–600; 0–1477)

At the time of assessments, 132 participants (96 %) rated their motor status as “on” or “on with dyskinesias” and 6 (4 %) rated it as “off”

FOGQsa, Freezing of Gait Questionnaire, self-administered version; H&Y, Hoehn and Yahr; NRT, Nutt Retropulsion test; PD, Parkinson’s disease; q1–q3, 1st–3rd quartile; SD, standard deviation; TG, Tandem gait; 10MWT, 10-Meter Walk test; m/s, meter per second; UPDRS item 30, Item 30 of Unified PD Rating Scale; UPDRS part III, motor score of the Unified PD Rating Scale

^a^Dichotomous question (Yes/No): *In the last 12* *months, have you fallen in such a way that your body hit the ground?*

^b^Dichotomous question (Yes/No): *Are you ever close to falling, but you manage to grab on to something/someone at the last minute so that your body does not hit the ground?*

^c^Scores ≥1 on the FOGQsa, item 3 (*Do you feel that your feet get glued to the floor while walking, making a turn or when trying to initiate walking (freezing)?*) were categorized as having FOG

^d^One missing value

^e^Two missing values

^f^Scores ≥1 on the NRT (unexpected shoulder pull) were categorized as having retropulsion

^g^Scores ≥1 on the UPDRS, item 30 (expected shoulder pull) were categorized as having retropulsion

^h^Scores ≥1 on the TG were categorized as abnormal

^i^Derived according to Tomlinson et al. [34]

**Table 2:** Proportion of individuals with/without falls/near falls based on 6-month follow-up, *n* = 138

Falls, *n* (%)	Near falls, but no falls, *n* (%)	Falls and/or near falls, *n* (%)	No falls or near falls, *n* (%)
45 (33)	19 (14)	64 (46)	74 (54)

Testing the ability of the 3-step model to distinguish between individuals with and without future falls yielded an AUC (95 % CI) of 0.74 (0.65–0.84). Further details are presented in Table 3. Considering the suggested risk categories [18], 55, 48 and 32 people scored in the low, moderate and high risk categories, respectively. Of these, there were 7 (13 %), 18 (38 %) and 20 (63 %) who actually fell during the subsequent 6 months.

**Table 3:** External validation of the 3-step model for prediction of future falls, *n* = 135

Predictors	Wald	*P* value	OR (95 % CI)
History of falls^a^	7.26	0.007	3.34 (1.39–8.02)
History of FOG^b^	0.79	0.376	1.48 (0.62–3.49)
Comfortable gait speed <1.1 m/s (10MWT)	6.02	0.014	2.88 (1.24–6.72)

Multiple logistic regression analysis (enter method); Nagelkerke pseudo *R* square: 0.229; Hosmer and Lemeshow test: *P* = 0.905; tolerance: ≥0.81 Sensitivity/specificity, 0.37/0.92

CI, confidence interval; FOG, Freezing of Gait; FOGQsa, Freezing of Gait Questionnaire, self-administered version; OR, odds ratio; 10MWT, 10-Meter Walk test; m/s, meter per second

^a^Dichotomous question (Yes/No): *In the last 12* *months, have you fallen in such a way that your body hit the ground?*

^b^Scores ≥1 on the FOGQsa, item 3 (*Do you feel that your feet get glued to the floor while walking, making a turn or when trying to initiate walking (freezing)?*) were categorized as having FOG

Simple logistic regression analyses of all available predictors and with falls as the dependent variable (Table 4) showed that near falls (AUC, 0.69) had the highest ability to distinguish between individuals with and without future falls. Rerunning these simple logistic regression analyses but with future falls and/or near falls as the dependent variable showed that a history of near falls and TG had the highest discriminant ability, both with an AUC (95 % CI) of 0.71 (0.62–0.80). Corresponding ORs (95 % CI) were 6.21 (2.91–13.25) for TG and 7.45 (3.32–16.70) for history of near falls (details available on request).

**Table 4:** Simple logistic regression analysis: prediction of future falls, *n* = 138

	OR (95 % CI)	AUC (95 % CI)
History of falls^a^	5.44 (2.43–12.15)***	0.68 (0.57–0.78)
History of near falls^b^	5.14 (2.38–11.10)***	0.69 (0.59–0.79)
History of FOG^c^, *n* = 137	3.1 (1.48–6.49)**	0.64 (0.54–0.74)
Comfortable gait speed <1.1 m/s (10MWT), *n* = 136	4.13 (1.92–8.85)***	0.67 (0.57–0.77)
Retropulsion (NRT)^d^	5.40 (2.39–12.20)***	0.67 (0.57–0.77)
Retropulsion (UPDRS item 30)^e^	2.52 (1.21–5.24)*	0.61 (0.51–0.71)
Abnormal tandem gait (TG)^f^	4.07 (1.81–9.17)**	0.66 (0.56–0.75)

AUC, area under the curve; CI, confidence interval; FOG, freezing of Gait; FOGQsa, freezing of Gait Questionnaire, self-administered version; NRT, Nutt Retropulsion test; OR, odds ratio; TG, Tandem gait; 10MWT, 10-Meter Walk test; m/s, meter per second; UPDRS item 30, Item 30 of unified Parkinson’s Disease Rating Scale

*** *P* < 0.001, ** *P* < 0.01, * *P* < 0.05

^a^Dichotomous question (Yes/No): *In the last 12* *months, have you fallen in such a way that your body hit the ground?*

^b^Dichotomous question (Yes/No): *Are you ever close to falling, but you manage to grab on to something/someone at the last minute so that your body does not hit the ground?*

^c^Scores ≥1 on the FOGQsa, item 3 (*Do you feel that your feet get glued to the floor while walking, making a turn or when trying to initiate walking (freezing)?*) were categorized as having FOG

^d^Scores ≥1 on the NRT (unexpected shoulder pull) were categorized as having retropulsion

^e^Scores ≥1 on the UPDRS, item 30 (expected shoulder pull) were categorized as having retropulsion

^f^Scores ≥1 on the TG were categorized as abnormal

Multiple logistic regression analysis (backward method) using all available predictors as independent variables resulted in three significant predictors for the occurrence of future falls (Table 5): history of near falls, retropulsion (NRT), and comfortable gait speed <1.1 m/s, with an AUC (95 % CI) of 0.78 (0.70–0.86). Rerunning this analysis using falls and/or near falls as the dependent variable identified three predictors with an AUC (95 % CI) of 0.82 (0.75–0.89). The three predictors were (OR; 95 % CI): history of near falls (5.08; 2.04–12.66), retropulsion (NRT) (3.40; 1.26–9.14), and TG (4.41; 1.91–10.19) (sensitivity/specificity, 0.57/0.86, tolerance, ≥0.87 details available on request).

**Table 5:** Extended multiple regression analysis: prediction of future falls, *n* = 135

Predictors^a^	Wald	*P* value	OR (95 % CI)
History of near falls^b^	6.33	0.012	3.03 (1.28–7.17)
Retropulsion (NRT)^c^	7.43	0.006	3.53 (1.43–8.72)
Comfortable gait speed <1.1 m/s (10MWT)	4.64	0.031	2.55 (1.09–5.98)

Multiple logistic regression analysis backward method (Wald); Nagelkerke pseudo *R* square: 0.299; Hosmer and Lemeshow test: *P* = 0.903; tolerance: ≥0.85

Sensitivity/specificity, 0.58/0.87

CI, confidence interval; FOGQsa, Freezing of Gait Questionnaire, self-administered version; NRT, Nutt Retropulsion test; OR, odds ratio; 10MWT, 10-Meter Walk test; m/s, meter per second

^a^Independent variables in the analysis were: history of falls past 12 months, history of near falls, history of FOG (FOGQsa item 3), comfortable gait speed <1.1 m/s (10MWT), retropulsion (NRT), retropulsion (UPDRS item 30), abnormal tandem gait (TG)

^b^Dichotomous question (Yes/No): *Are you ever close to falling, but you manage to grab on to something/someone at the last minute so that your body does not hit the ground?*

^c^Scores ≥1 on the NRT (unexpected shoulder pull) were categorized as having retropulsion

## Discussion

In this prospective study of individuals with relatively mild PD we externally validated the accuracy of a previously suggested 3-step model for prediction of falls [18, 19]. We found the discriminant ability of this model to be lower but acceptable and overlapping (given the 95 % CIs of AUCs) compared to previous studies [18, 19]. Importantly, discriminant abilities of each single predictor were lower and below acceptable values. This supports the value of the 3-step model over reliance on single predictors.

Different study samples have revealed some differences regarding the contribution of each predictor in the 3-step model. For example, in the development study [18], FOG was significant and associated with more than a two-fold increased odds of falling, while it was not significant in the subsequent [19] or in our study despite similar percentages of individuals reporting FOG (41–46 %) in all three samples. These discrepancies may be due to methodological aspects, as FOG was not assessed uniformly across the studies; Paul et al. [18] specified a retrospective time frame of 1 month, whereas both Duncan et al. [19] and we used a dichotomized version of item 3 of the FOGQ, which does not specify the recall period. Furthermore, information on history of FOG does not take FOG severity into account [38]. Similarly, differences in observed ORs for history of falls may relate to different modes of data collection. Specifically, whereas we and Paul et al. [18] inquired about the presence or absence of falls during the past year, Duncan et al. [19] combined data from two time points 6 months apart, where a question with five response categories was used.

Regardless of the cause(s) for the observed discrepancies in ORs of individual predictors, this has implications for the suggested scoring weights and risk categories of the 3-step model. That is, the weights (scores) suggested by Paul et al. [18] were based on the observed ORs in that study, which have not been replicated either here or by Duncan et al. [19]. It can be noted that the percentages of individuals who actually fell in our study was 13, 38 and 63 % in the low, moderate and high risk categories, respectively. Corresponding values in the study by Duncan et al. were 9, 28 and 66 % [19]. This is in contrast to the expected probabilities suggested by Paul et al. (17, 51 and 85 %, respectively) [18]. This calls for caution regarding the use of the suggested weighted total score. Further studies are needed to firmly establish a scoring system and risk categories.

The contribution of gait speed was relatively similar here as compared to the study by Paul et al. [18], despite differences in motor status according to the UPDRS part III (12 vs. about 24). Thus, comfortable gait speed <1.1 m/s is associated with approximately a two-fold increase in odds of falling regardless of whether a 4- [18] or 10-meter walking distance was used. This suggests the possibility to adjust the walking distance according to practical circumstances. However, the need to calculate the mean value for two trials should be evaluated in order to explore the possibility to simplify the test.

According to current recommendations regarding improvement of prediction models [17, 19] we explored the addition of history of near falls, retropulsion, and TG to the 3-step model. This generated a new model including history of near falls, retropulsion (NRT) and gait speed. The discriminate ability of this new model as well as its sensitivity of prediction was somewhat better compared to the proposed 3-step model but the AUC 95 % CIs overlapped. Similarly, using falls and/or near falls as the dependent variable generated a model including history of near falls, retropulsion (NRT) and TG. These observations have important clinical implications. Near falls are more frequent than falls in PD [39, 40] and may occur also among those who do not experience falls [39, 41]. We previously found, in the same project, that history of near falls but not falls was a risk factor for future falls [12]. This is further supported here and suggests that information about near falls may be a useful predictor of future falls. Furthermore, since near falls may be seen as an early precursor of increased fall risk [42, 43], it is argued that prediction of falls and/or near falls has greater clinical value than prediction of falls alone. This is also in line with previous studies highlighting the importance of fall risk identification before the first fall has occurred, in order to optimize planning of interventions [9, 16]. From this perspective, our new model (history of near falls, TG and retropulsion according to the NRT) may be considered a promising alternative to the suggested 3-step model, at least among people with milder PD. Indeed, the use of TG and NRT has been recommended in the prediction of falls before [23, 24, 44]. However, this suggested new model needs further confirmation in additional studies.

NRT, but not UPDRS item 30 was identified as a predictor in both new models. UPDRS item 30 involves prior instructions, which does not mimic daily life circumstances where perturbations per definition are unexpected [44]. Accordingly, the unexpected pull test according to the NRT has been considered more relevant in the context of fall prediction [24], which is supported by our findings.

### Limitations

This study involves people with relatively mild PD, excluding those with MMSE scores <24 or >80 years old. This limits the generalizability of findings, particularly regarding predictors explored in addition to the suggested 3-step model. Further studies are therefore needed to explore the external validity of these models in broader ranges of PD severities. Particularly, larger longitudinal studies addressing near falls and TG are needed to better understand these variables in the context of falls prediction.

Furthermore, we acknowledge that there might be other questions, questionnaires and clinical assessments that also may be of relevance in relation to fall prediction [9, 16, 45]. Finally, we did not consider the influence of the suggested 3-step model or other identified models on decision making, patient outcomes, or costs [17]. This will need to be addressed in specifically designed studies.

## Conclusions

This study confirms the value of the 3-step model as a clinical fall prediction tool and illustrates that it outperforms the use of single predictors. However, further studies are needed to firmly establish a scoring system and risk categories based on this model, and to better understand the influence of methodological aspects of data collection regarding gait speed and history of falls and FOG. A new model for prediction of falls and near falls, including history of near falls, TG and retropulsion according to the NRT is considered a promising alternative to the 3-step model in milder PD, but needs to be tested in additional samples. Taken together, our observations provide important additions to the evidence base for clinical fall prediction in PD.
